# Spatiotemporal parameters and gait variability in people with psoriatic arthritis (PsA): a cross-sectional study

**DOI:** 10.1186/s13047-022-00521-y

**Published:** 2022-03-04

**Authors:** Roua Walha, Nathaly Gaudreault, Pierre Dagenais, Patrick Boissy

**Affiliations:** 1 Faculty of Medicine and Health Sciences, Université de Sherbrooke, Sherbrooke, QC Canada; 2 Research Center on Aging, CIUSSS Estrie CHUS, Sherbrooke, QC Canada

**Keywords:** Psoriatic arthritis, Foot pain, Foot function, Spatiotemporal parameters, Gait variability, Gait analysis

## Abstract

**Background:**

Foot involvement is a major manifestation of psoriatic arthritis (PsA) and can lead to severe levels of foot pain and disability and impaired functional mobility and quality of life. Gait spatiotemporal parameters (STPs) and gait variability, used as a clinical index of gait stability, have been associated with several adverse health outcomes, including risk of falling, functional decline, and mortality in a wide range of populations. Previous studies showed some alterations in STPs in people with PsA. However, gait variability and the relationships between STPs, gait variability and self-reported foot pain and disability have never been studied in these populations. Body-worn inertial measurement units (IMUs) are gaining interest in measuring gait parameters in clinical settings.

**Objectives:**

To assess STPs and gait variability in people with PsA using IMUs, to explore their relationship with self-reported foot pain and function and to investigate the feasibility of using IMUs to discriminate patient groups based on gait speed-critical values.

**Methods:**

Twenty-one participants with PsA (age: 53.9 ± 8.9 yrs.; median disease duration: 6 yrs) and 21 age- and sex-matched healthy participants (age 54.23 ± 9.3 yrs) were recruited. All the participants performed three 10-m walk test trials at their comfortable speed. STPs and gait variability were recorded and calculated using six body-worn IMUs and Mobility Lab software (APDM®). Foot pain and disability were assessed in participants with PsA using the foot function index (FFI).

**Results:**

Cadence, gait speed, stride length, and swing phase were significantly lower, while double support was significantly higher, in the PsA group (*p* < 0.006). Strong correlations between STPs and the FFI total score were demonstrated (|r| > 0.57, *p* < 0.006). Gait variability was significantly increased in the PsA group, but it was not correlated with foot pain or function (*p* < 0.006). Using the IMUs, three subgroups of participants with PsA with clinically meaningful differences in self-reported foot pain and disability were discriminated.

**Conclusion:**

STPs were significantly altered in participants with PsA, which could be associated with self-reported foot pain and disability. Future studies are required to confirm the increased gait variability highlighted in this study and its potential underlying causes. Using IMUs has been useful to objectively assess foot function in people with PsA.

**Trial registration:**

ClinicalTrials.gov, NCT05075343, Retrospectively registered on 29 September 2021.

## Background

Psoriatic arthritis (PsA) is a chronic inflammatory arthropathy associated with skin psoriasis and belongs to the spondyloarthropathy family. Several musculoskeletal manifestations can occur during the disease course, and both axial and peripheral joints can be affected. The foot and the ankle are common targets of inflammation and their involvement could be a major manifestation of the disease in terms of frequency and severity [1–3]. Foot and ankle problems include dactylitis, enthesitis, synovitis, and tenosynovitis and can lead to foot and/or ankle pain, stiffness, swelling, and deformity [4–8]. Consequently, a high proportion of patients could experience moderate to high levels of foot impairment and difficulties with activities of daily living that require good foot function, such as walking [3, 4, 9].

Pain and physical function are identified among the most important clinical domains to be measured in PsA clinical studies by the Group for Research and Assessment of Psoriasis and Psoriatic arthritis (GRAPPA) [10]. While patient-reported outcomes are commonly used to assess pain and perceived function, gait analysis could be used to obtain objective measures of physical function [11] In people with inflammatory joint disease, including rheumatoid arthritis (RA) and PsA, different gait parameters of varying complexity, such as joint kinetics and kinematics, plantar pressure, and spatiotemporal parameters (STPs), have been employed to assess either global function or localized foot function [12–14]. Among these parameters, STPs, which typically encompass gait speed, stride length, cadence, double support, and swing time present certain ease of interpretability by both clinicians and patients and have great utility in predicting health outcomes. For example, a reduced gait speed was associated with an increased risk of falling [15], functional decline [16] and mortality in older adults [17]. Gait speed was also designated the 6th vital sign, and precise cut-off values have been used to predict specific outcomes in older adults [18, 19]. Both the STP mean values and the variation around them, referred to as gait variability, are key metrics in gait evaluation [20]. Gait variability is used as a clinical index for gait stability [21] and is associated with an increased risk of falling in older adults [22].

Importantly, STPs and gait variability can now easily be measured with emerging lightweight, low-cost, and easy-to-use wearable inertial measurement units (IMUs). These latter have shown acceptable accuracy and precision in measuring STPs in people with PsA and axial spondyloarthritis [23, 24].

Many studies investigated gait STPs in people with RA with foot involvement and showed significant alterations in gait STP which included reduced gait speed, stride length and cadence, and increased double support [14]. However, there are scarce data on gait STPs in people with spondyloarthritis, including PsA [14, 25]. For instance, a recent study demonstrated changes in gait STPs including reduced gait speed, stride length, and swing time, and increased double support time, which were associated with self-reported pain in people with axial spondyloarthritis [26]. Similar changes were reported in a few studies in people with PsA [5, 27, 28]. For example, Hyslop et al. assessed cadence, gait speed, stride length, and double support time in people with PsA with and without enthesitis and showed that stride length was significantly lower in the PsA group with enthesitis [27]. A study by Woodburn et al., which was based on the same cohort as Hyslop et al., showed a significant decrease in gait speed in a PsA group with enthesitis compared to healthy controls [28]. On the other hand, Wilkins et al. investigated cadence, gait speed, and double support time in people with PsA with and without active dactylitis. Their findings showed a decreased gait speed and increased double support in both PsA groups. However, no significant differences were demonstrated compared to the control group. This could be explained by the small sample size, the relatively young mean age of the study participants (36.7 ± 21.5 years) and the short disease duration (4.6 ± 6.7 years), which were previously shown to be correlated with gait parameters in people with RA [5, 29].

Overall, the above studies demonstrate alterations in gait STPs. However, despite including participants with confirmed foot involvement, it is not clear whether altered gait STPs are associated with self-reported foot pain and disability. On another note, the reported alterations could be indicative of increased gait instability since such changes are characteristics of cautious gait patterns that are typically undertaken by older adults to increase stability [30]. In fact, a few recent studies demonstrated altered static and dynamic balance [31, 32] and increased risk of falling in people with PsA [33]. Fall-related risk factors have not been studied in people with PsA. Nevertheless, research in RA showed that swollen and tender lower extremity joints were among the most significant fall-related risk factors [34]. Taking all this into account, despite being a relevant and easy-to-measure gait parameter, no previous research has investigated gait variability and its relationship with self-reported foot pain and function in people with PsA.

Thus, given the limited evidence regarding STPs, gait variability, and their relationship with foot pain and disability in people with PsA, this study aimed 1) to investigate STPs and gait variability in participants with PsA with foot pain and compare them to age- and sex-matched healthy participants using body-worn IMUs, 2) to explore the relationship between STPs, gait variability, and self-reported foot pain and disability, and 3) to investigate the feasibility of using body-worn IMUs to discriminate patient groups based on gait speed-critical values.

## Methods

### Study design

A portion of the data presented in this descriptive cross-sectional study pertains to an ongoing pre-experimental trial exploring the effects of custom-made foot orthoses on foot pain and function and gait STPs in people with PsA. Baseline gait STP measures in participants with PsA captured during a standardized 10-m walk within this pre-experimental study were compared to age- and sex-matched controls undergoing the same clinical gait evaluation protocol.

### Participants

Twenty-one participants with PsA were consecutively recruited from the rheumatology outpatient clinics at the Hotel Dieu University Hospital CHU of Sherbrooke (CHUS). Inclusion criteria were the following: being between 20 and 70 years of age, having a rheumatologist-confirmed PsA diagnosis, having recurrent and moderate (> 3 points) to severe (> 6 points) foot pain as reported on a 0 to 10 numerical rating scale [35], and receiving stable medication for at least the three months preceding recruitment. Exclusion criteria applied to patients with diabetes, neurological disease, or any musculoskeletal disease that could impact normal gait patterns. Patients who received intra-articular corticosteroid injections or any conservative foot treatment, such as foot orthoses, within the past three months were excluded because they may influence their gait. Twenty-one control participants matched for age and sex with no self-reported foot/ankle problems were also recruited using flyers posted in the research center and the word-of-mouth strategy. They had to be devoid of a current or recent history of foot/ankle pain and self-reported gait deficits. The study was approved by the CIUSSS de l’Estrie-CHUS Institutional Review Board and all the participants provided written informed consent.

### Data collection procedure

Upon their arrival at the Université de Sherbrooke Research Center on Aging, demographic data, including sex, age, body mass index (BMI), and foot and lower limb pain, were obtained for all participants (PsA and controls). Perceived foot function was additionally assessed in PsA participants. Afterward, an instrumented gait analysis was performed for each participant (PsA and controls). Disease-related information was obtained for participants with PsA from their medical records.

### Outcomes and measurement tools

#### Clinical parameters

##### Disease characteristics

Disease duration, current medication, and C-reactive protein (CRP) levels, as a marker of systemic inflammation, were obtained from the patient’s medical record.

##### Foot and lower limb pain

Given that lower limb and lower back pain are not uncommon in people with PsA and that they could affect gait patterns [26, 36–39], knee, hip, and lower back pain were assessed in addition to foot pain using the numerical rating scale (NRS) in participants with PsA and healthy controls. Participants were asked to circle a number between 0 and 10 that best fits their average pain intensity experienced in the foot, knee, hip, and lower back over the seven days preceding the data collection. Moreover, all the participants in the PsA group were examined by a trained podiatrist with eight years of professional clinical experience, in an independent podiatry clinic. Pain sites and deformities at the feet were documented from the podiatrist’s clinical examination record.

##### Foot function

Foot function was measured in the PsA group with the Foot Function Index (FFI), a reliable and valid questionnaire that has been proven suitable for use in people with foot disorders and a low functioning status [40, 41]. The FFI was chosen to be used in this study because it has a validated version in French [42]. The FFI comprises 23 items divided into three sub-scales measuring foot pain (FFI-Pain), foot disability (FFI-Disability), and foot-related activity limitation (FFI-Activity Limitation). Each FFI item is recorded on an NRS (0 to 10). A total score and three sub-scale scores were calculated. For an easier interpretation, the scores are presented as percentages, where a higher percentage indicates higher levels of foot pain and related disability.

#### Gait analysis

**Fig. 1:**
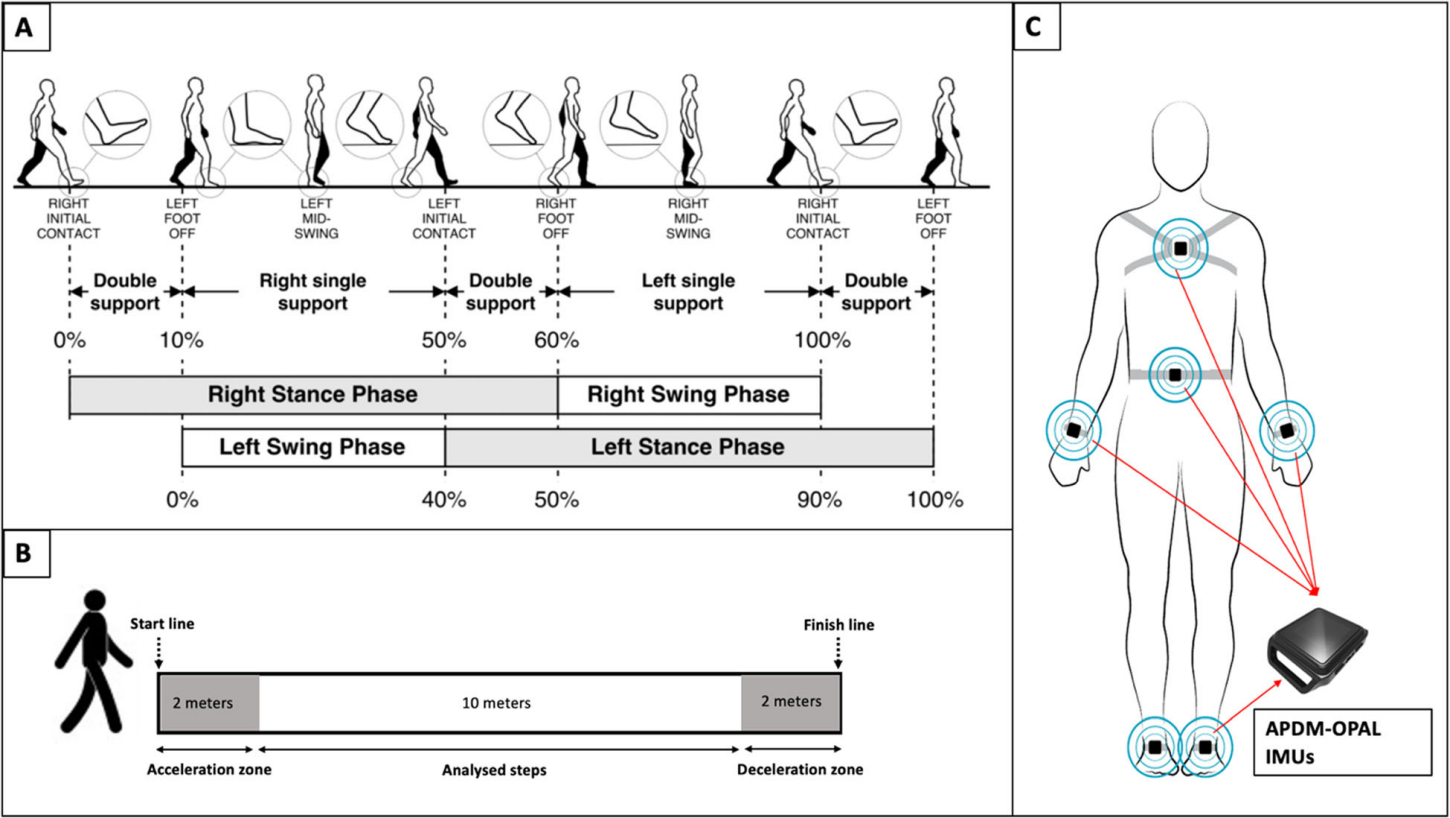
**A** The gait cycle phases taken from an open access article [40]; **B** 10 m walk test (10MWT); **C** The Mobility Lab sensors placement

Gait STPs presented and defined in Table 1 and Fig. 1A [44] were measured in participants with PsA and healthy controls using Opal IMUs and Mobility Lab software (APDM Wearable Technologies, Portland, OR, USA). The Mobility Lab has been validated in healthy and pathological populations such as Parkinson’s disease [45–47]. The accuracy of this system was also assessed in people with PsA in a previous work that showed acceptable errors in measuring gait STPs recorded over a treadmill at a normal walking speed [23]. The mobility lab includes a set of six IMUs each with a triad of sensors (a 3-axis accelerometer, a 3-axis gyroscope, and a 3-axis magnetometer) (Fig. 1. C), an access point for wireless data transmission and synchronization and software (Mobility Lab) that provides an automated estimation of several STPs (Table 1). Details on the algorithm allowing for STP calculation with the Mobility Lab system have been previously described [48].

**Table 1:** Gait Spatiotemporal Parameters (SPTs), measurement units, and definitions

Variables	Units	Definitions
**Cadence**	Step/minute (step/min)	Number of steps per minute
**Gait speed**	Meter/second (m/s)	The forward speed of the subject, measured as the forward distance traveled during the gait cycle divided by the gait cycle duration.
**Stride length**	Meters (m)	The forward distance traveled by a foot during a gait cycle.
**Double support time**	% GCT	The percentage of the gait cycle in which both feet are on the ground.
**Swing time**	% GCT	The percentage of the gait cycle in which the foot is not on the ground.
**Foot strike angle**	degrees	The angle of the foot dorsiflexion at the point of initial contact. The pitch of the foot when flat is zero and positive when the heel contacts first.
**Stride time variability**	%	The percentage of each participant’s standard deviation of stride time is divided by the same parameter mean value.

*GCT* gait cycle time

Definitions were provided by [43]

All the participants performed three trials of the 10-m walk test (10MWT) (Fig. 1. B), which consists of walking over a 14-m straight walkway at a comfortable speed. Two extra meters at the beginning and the end of the trials were added to account for the acceleration and deceleration, and only the central 10 m were considered for the analysis (Fig. 1. B). The participants were asked to walk at their usual preferred self-selected speed wearing comfortable walking shoes, and none of the participants used foot orthoses or modified footwear. The Mobility Lab IMUs were fixed with elastics straps on the chest, the lower back, and both wrists and feet as recommended by the manufacturer’s instructions (Fig. 1. C).

Stride time variability was chosen as a measure of gait variability, as it is the most commonly reported parameter in clinical studies [49]. This metric was calculated as the coefficient of variation (CV), defined as the percentage of each participant’s standard deviation of stride time divided by its mean value:

$$ \mathrm{CV}=\frac{\ \mathrm{SD}}{\mathrm{Mean}}\ \mathrm{x}\ 100\% $$

### Statistical analysis

Based on data from a previous study comparing gait parameters between people with PsA diagnosed with rearfoot enthesitis and healthy controls [27], an average effect size was calculated from the means and standard deviations reported for cadence, gait speed, stride length, and double support time. Given the calculated effect size (d = 0.8), a total sample size of 32 participants (16 per group) was required to detect significant differences in gait STPs with a paired t-test at an α of 0.01 and a power of 0.95.

The Shapiro-Wilk test was used to examine data distribution. Paired t-tests and the Wilcoxon signed-rank test were used to assess the differences in gait STPs and stride time variability between participants with PsA and matched healthy controls, and Cohen’s effect size was calculated to quantify the magnitude of these differences. ANCOVA was used to adjust the differences in STPs between PsA and healthy participants for the effect of BMI. Pearson’s and Spearman’s correlation coefficients were calculated to assess the relationships between STPs, gait variability, and self-reported foot pain and function in participants with PsA. Correlation coefficients were considered weak, moderate and strong for values between 0.1 and 0.3, 0.3 and 0.5, and > 0.5, respectively [50]. Given the clinical relevance of gait speed and the availability of reference values for this metric, subgroups of participants with PsA were differentiated based on critical values of gait speed (1.0 m/s: the limit below which gait speed values are associated with higher mortality and 1.2 m/s: the lower limit of the confidence interval for normative gait speed [17, 51]). The relationships between gait speed, FFI total score and FFI sub-category scores for these three subgroups were visualized in scatter plots.

As multiple variables that may be highly correlated were tested, the Bonferroni correction method was used to reduce type I error resulting from multiple testing. Therefore, *P* values < 0.006 were considered statistically significant. Analyses were performed using SPSS version 26.0 (IBM SPSS, Armonk, NY).

## Results

### Demographics and clinical characteristics

Twenty-one participants with PsA (5 males, 16 females) with a mean age of 53.9 ± 8.9 years and a mean disease duration of 11.5 ± 10.2 years and 21 healthy controls (5 males, 16 females) with a mean age of 54.2 ± 9.3 years were included (Table 1). BMI was significantly higher in participants with PsA than in healthy controls (29.3 ± 4.5 vs 24.4 ± 3.4), *p* < 0.001). Because of the different thresholds used by the laboratories, CRP levels are reported as normal or high. CRP levels were missing, high and normal for 4, 1 and 16 participants, respectively. Ninety percent of the patients were treated with disease-modifying anti-rheumatic drugs (DMARDs) and/or biological therapy.

The NRS scores showed moderate to severe levels of lower limb pain in the PsA group. 52.4 and 38.1% of the participants with PsA reported moderate and severe foot pain, respectively, while foot and lower limb pain levels were close to zero in healthy participants (Table 2). The FFI sub-scores also showed moderate to severe levels of self-reported foot pain (55.7 ± 18.3) and disability (44.6 ± 22.7) in participants with PsA. The most frequently reported pain sites at the feet were the ankles, followed by the metatarsals, toes, and heels (plantar and/or posterior heel), and 18 (85%) participants had simultaneous forefoot and rearfoot pain. Sixty-two percent of the participants in the PsA group had heel valgus, 67% had hammer/claw toes and 24 and 19% had hallux valgus and hallux rigidus, respectively.

**Table 2:** Demographics and clinical characteristics of participants with PsA and healthy participants

Variables	PsA	CONTROLS
	Mean ± SD	Mean ± SD
**AGE (years)**	53.9 ± 8.9	54.23 ± 9.3
**BMI (kg/m**^**2)**^	29.3 ± 4.5)	24.4 ± 3.4*
**SEX (M**: **F)**	5: 16	5: 16
**DISEASE DURATION (years)**	11.5 ± 10.2 (Median = 6, IQR: 12)	–
**CRP (mg/l)**		
**Normal**	16 (94%)	
**High**	1 (6%)	
**Pharmacological therapy**		
**DMARDs**	6 (30%)	
**Biological therapy**	5 (25%)	
**DMARDs and Biological therapy**	7 (35%)	
**Foot pain (0 to 10 points)**	5.6 ± 1.9	0.2 ± 0.6*
**Knee pain (0 to 10 points)**	4.7 ± 2.6	0.5 ± 1.2*
**Hip pain (0 to 10 points)**	4.8 ± 2.9	0.1 ± 0.3*
**Lower back pain (0 to 10 points)**	5.4 ± 2.7	1.6 ± 2.5*
**Foot function Index**		
**FFI-Pain (%)**	55.7 ± 18.3	–
**FFI-Disability (%)**	44.6 ± 22.7	
**FFI-Activity limitation (%)**	34.3 ± 24.4	
**FFI-Total (%)**	47.02 ± 18.3	
**Pain sites**		–
**Toes**	15 (71%)	
**Metatarsals**	16 (76%)	
**Heels**	11 (52%)	
**Ankles**	17 (81%)	
**Deformities**		–
**Rearfoot valgus**	13 (62%)	
**Hallux valgus**	5 (24%)	
**Hallux rigidus**	4 (19%)	
**Hammer/claw toes**	14 (67%)	

Values are mean ± standard deviation and percentages for categorial variables, *p* values < 0.006 are considered significant, *: *p* < 0.006. *BMI* body mass Index, *M* males, *F* females, *FFI* foot function index

### Spatiotemporal parameters and gait variability in PsA and healthy participants

Gait STPs for left and right foot in PsA and healthy participants are presented in Table 3. There were significant differences between the two groups in all the measured STPs except for the foot strike angle of the right foot. STPs averaged for left and right foot and stride time variability adjusted for BMI in PsA and healthy participants are summarized in Table 4. Before adjusting the data for BMI, all STPs except for foot strike angle were significantly different between groups. Cadence, gait speed and stride length and swing time were significantly lower in participants with PsA, and large effect sizes were reported (*p* < 0.006; 1.08 < d < 1.3) (Table 4). Gait cycle duration and double support time were significantly higher in the PsA group, and large effect sizes were also reported (*p* < 0.006; d = 1.2 and d = 1.26) (Table 4). After adjusting the differences for BMI, only cadence, gait cycle duration, and gait speed remained significantly different between groups, while the differences in stride length (*p* = 0.026), double support (*p* = 0.022), and swing time (*p* = 0.023) were no longer significant. Stride time variability was significantly higher in the PsA group before and after adjusting for BMI (*p* = 0.001 and *p* = 0.005), and a moderate effect size was reported (d = 0.68) (Table 4).

**Table 3:** Spatiotemporal parameters (STPs) for left and right foot in participants with PsA and healthy matched controls

Variable	PsA participants		Control participants		***p***-value	
	Left	Right	Left	Right	Left	Right
**Cadence (step/min)**	108.1 ± 10.8	107.7 ± 10.8	120 ± 6.8	119.9 ± 6.9	0.000	0.000
**Gait cycle duration (s)**	1.1 ± 0.1	1.1 ± 0.1	1 ± 0.1	1 ± 0.1	0.000	0.000
**Gait speed (m/s)**	1.1 ± 0.2	1.1 ± 0.2	1.4 ± 0.2	1.3 ± 0.2	0.000	0.000
**Stride length (m)**	1.2 ± 0.2	1.2 ± 0.2	1.4 ± 0.2	1.3 ± 0.1	0.001	0.001
**Double support time (% GCT)**	21.9 ± 4.1	22.1 ± 4.1	17.8 ± 2.7	18 ± 2.7	0.000	0.000
**Swing time (% GCT)**	39.4 ± 1.9	38.6 ± 2.4	41.3 ± 1.4	40.8 ± 1.4	0.001	0.000
**Foot strike angle (degrees)**	25.3 ± 4.1	24.7 ± 3.9	28.6 ± 2.7	27.7 ± 3.7	0.008	0.015

Values are Mean ± standard deviation, *p* values < 0.006 are considered significant

*PsA* Psoriatic arthritis, *GCT* gait cycle time

**Table 4:** Spatiotemporal parameters (STPs) averaged for left and right foot and stride time variability in participants with PsA and healthy matched controls before and after adjustment for BMI

Variables	PsA participants		Control participants		Cohen’s d
	Mean ± SD	Adj Mean (SE)	Mean ± SD	Adj Mean (SE)	
**Cadence (step/min)**	107.91 ± 10.78	107.47 (2.12)	120.08 ± 6.8^a^	120.55 (2.17) ^b^	1.3
**Gait cycle duration (s)**	1.13 ± 0.14	1.13 (0.03)	1.00 ± 0.06^a^	1.00 (0.03) ^b^	1.2
**Gait speed (m/s)**	1.07 ± 0.23	1.10 (0.05)	1.35 ± 0.2^a^	1.38 (0.06) ^b^	1.3
**Stride length (m)**	1.17 ± 0.18	1.19 (0.04)	1.34 ± 0.13^a^	1.32 (0.04)	1.08
**Double support (% GCT)**	22.00 ± 4.13	21.43 (0.79)	17.95 ± 2.7^a^	18.55 (0.81)	1.16
**Swing time (% GCT)**	39.0 ± 2.08	39.28 (0.40)	41.03 ± 1.36^a^	40.73 (0.41)	1.15
**Foot strike angle (degrees)**	24.99 ± 3.79	25.16 (0.80)	28.15 ± 2.97^a^	27.97 (0.82)	0.92
**Stride time variability (%)**	4.03 ± 3.56	4.49 (0.58)	2.32 ± 0.72^a^	1.84 (0.60) ^b^	0.68

Values are mean ± standard deviation and adjusted mean (Standard error)

*PsA* Psoriatic arthritis, *Adj* adjusted mean, *SE* Standard error, *d* Cohen’s effect size, *GCT* gait cycle time

^a^Significant differences in mean values between PsA and healthy participants

^b^Significant differences in adjusted mean values between PsA and healthy participants

Three subgroups of participants with PsA (PsA1, PsA2, and PsA3) were differentiated based on gait speed-critical values. Patients in PsA1 had gait speed values below 1.0 m/s with a mean gait speed of 0.81 ± 0.16 m/s; PsA2 included patients with gait speed values between 1.0 m/s and 1.2 m/s, and the mean gait speed for this subgroup was 1.08 ± 0.05 m/s; and PsA3 was composed of patients with gait speed values greater than 1.2 m/s with a mean gait speed of 1.34 ± 0.1 m/s. PsA1, PsA2 and PsA3 represented 32, 41 and 27% of the total sample, respectively.

### Relationship between STP, gait variability, and clinical parameters

**Fig. 2:**
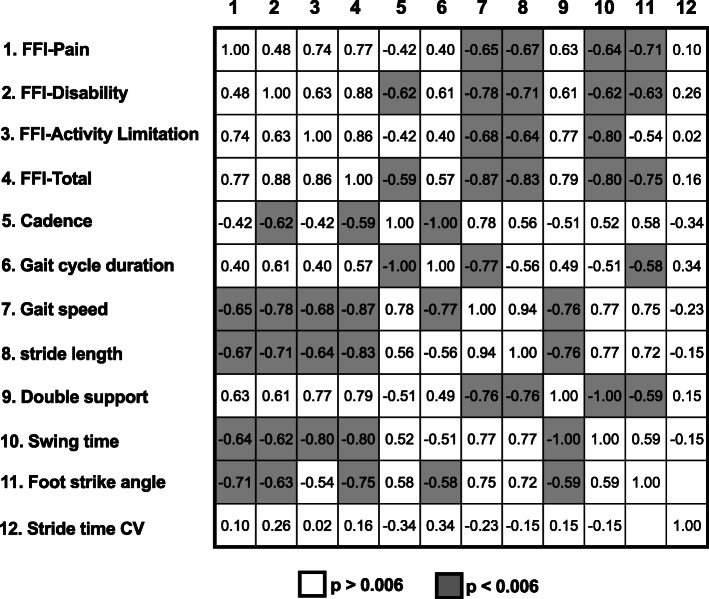
Correlation matrix of the relationships between spatiotemporal parameters, stride time variability and the foot function index

Correlation coefficients between STPs, stride time variability and clinical parameters in participants with PsA are presented in Fig. 2. All the STPs were strongly and significantly correlated with the FFI total score and the disability sub-score (0.57 < |r| < 0.87, *p* < 0.006). All the STPs except for cadence and gait cycle duration were correlated with the pain and activity limitation sub-scores (0.63 < |r| < 0.8, *p* < 0.006) (Fig. 2). Gait speed had the highest correlation coefficients with the FFI total score (r = − 0.87) and the disability sub-score (r = − 0.78), while foot strike angle had the highest correlations with the pain sub-score (FFI-Pain) (r = − 0.71). Swing time (r = − 0.80) had the highest correlations with the activity limitation sub-score (FFI-Activity limitation). Regarding gait variability, there was no significant correlation between stride time CV and clinical parameters of foot pain and disability.

**Fig. 3:**
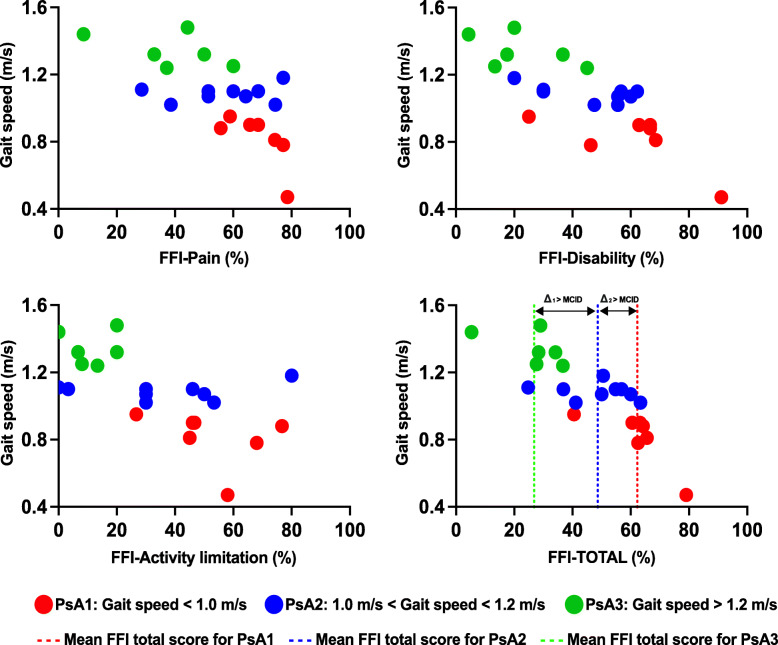
Scatter plots of the relationships between gait speed and the foot function index for PsA subgroups. *PsA1* Participants with gait speed below 1.0 m/s, *PsA2* participants with gait speed comprised between 1.0 and 1.2 m/s and *PsA3* participants with gait speed higher than 1.2 m/s, *FFI* Foot function index. Δ_1_ Difference in the FFI total score between PsA2 and PsA3. Δ_2_ Difference in the FFI total score between PsA1 and PsA2

The relations between gait speed and the total FFI score and sub-scores for PsA subgroups (PsA1, PsA2, and PsA3) are presented in Fig. 3. Participants in PsA1 had the highest scores on all the sub-scales and the total FFI compared to PsA2 and PsA3 (FFI-Total: 62.21 ± 11.36% for PsA1, vs 48.68 ± 12.36% and 26.83 ± 11.14% for PsA2 and PsA3, respectively). Knowing that the minimal clinically important difference (MCID) for the FFI total score is equal to 7%, the differences in the FFI total scores found between these subgroups were clinically meaningful.

It was theorized that CRP levels, disease duration, knee pain, hip pain, and lower back pain would affect STPs and interfere with their relationship with the FFI. However, none of these potential confounders was correlated with STPs except for knee pain, which was moderate with foot strike angle (r = − 0.48, *p* = 0.028), but the strength of the relationship did not reach the significance level.

## Discussion

The aims of this study were first to assess the differences in gait STPs and gait variability measured with IMUs during a 10-m walk test between participants with PsA and foot pain and age- and sex-matched healthy participants and second to investigate the relationships between gait STPs and variability and clinical outcomes of foot pain and disability.

### Spatiotemporal parameters

Our findings showed significant differences in all the STPs between participants with PsA and matched controls. These differences included lower cadence, gait speed, stride length, swing time, and foot strike angle and higher gait cycle duration and double support time in the PsA group than in the healthy controls. However, only cadence, gait speed, and gait cycle duration remained significantly different after adjusting for BMI. Nearly 50% of our PsA sample had a BMI above 30 kg/m^2^, which is not surprising because obesity is a common comorbidity of PsA [52]. Moreover, obesity is known to alter STPs, which has been suggested to be a strategy to lower joint loadings [53]. Therefore, it is logical that BMI affected the differences in STPs between participants with PsA and controls in our study.

A few previous studies showed some alterations in STPs in people with PsA, but not all of them demonstrated significant differences between PsA participants and healthy controls. It is important to mention that all these studies included participants with a younger mean age compared to that reported in our study. Of note, in a recent systematic review, age was shown to have significant effects on slowing STPs in healthy adults [30]. Thus, the more significant between-group differences demonstrated in the present study could be attributed to a combined effect of age and disease. Moreover, in the study by Hyslop et al., the participants were matched for BMI, which was normal in PsA and control participants [27]. The results reported in our study highlighted the effects of BMI on STP. Moreover, the impact of obesity on foot function and structure in older adults has been demonstrated in a previous study [54]. This suggests that increased BMI could significantly alter foot function and gait in people with PsA which could explain the nonsignificant differences reported in Hyslop. In addition, in this latter study, even though patients with confirmed enthesitis were included, low to moderate levels of foot pain were reported by the authors which can also help explain their findings. In our study, although nearly 90% of the PsA participants were managed on DMARDs/biologicals and most of them had normal CRP levels, a high prevalence of simultaneous forefoot and rearfoot pain and moderate to severe levels of self-reported foot pain and disability were demonstrated. This finding suggests that even though pharmacological treatments might be efficient on systemic inflammation control, significant foot pain and related disability can still be present in people with PsA.

Furthermore, clinically important differences in STPs between PsA and healthy participants and strong correlations between foot pain, foot function, and STPs, especially gait speed, were also demonstrated. Interestingly, these correlations were not affected by the CRP levels, disease duration, or lower limb pain since none of these clinical parameters was significantly correlated with STP. Although direct comparison between pain levels reported in Hyslop et al. and those reported in the present study cannot be made due to the different measurement tools used, our findings suggest that foot pain may play a major role in gait alterations in people with PsA.

Based on gait speed values, it was possible to discriminate between three PsA subgroups. PsA participants who had gait speed values below 1.0 m/s had higher FFI scores than those for whom gait speed was between 1.0 m/s and 1.2 m/s and those with gait speed above 1.2 m/s. There was not enough power to statically test the differences in the FFI scores between these three subgroups. However, knowing that the MCID for the FFI total score is 7 points, the results showed that differences between these gait speed-based subgroups could be clinically significant. This suggests that gait speed may be a relevant metric not only to assess gait alteration in people with PsA but also to have more objective insight into the impact of the disease on self-reported foot pain and disability.

The results from studies addressing gait STPs in patients with RA are coherent with our study. For example, a previous systematic review on gait analysis of the lower limb in patients with RA showed that they tend to walk slower, with a longer gait cycle, a shorter step length, a longer double support time, and a lower cadence compared to healthy subjects [55]. These findings were confirmed in a recent meta-analysis that reported a significant decrease in gait speed, stride length, and cadence and a significant increase in double support in patients with RA compared to healthy participants. Similar to the present study, this meta-analysis also reported large effect sizes for the differences between RA and healthy participants (effect sizes (95% CI) were 1.55 (0.83 to 2.27); 1.66 (1.49 to 1.84); 0.97 (0.45 to 1.49)) and 1.01 (0.66 to 1.36) for gait speed, stride length, cadence and double support time, respectively [14].

It appears that walking slower with shorter steps is a common compensatory strategy that people with arthritic foot disease use to reduce loads and pain in the affected joints and to increase stability [53, 56, 57]. It has been reported that reducing gait speed leads to lower joint flexion and extension moments in hip, knee, and ankle joints [58] and that reducing step length allows for a decrease in the vertical ground reaction forces [59–61]. Moreover, double limb support, in contrast to single limb support and swing (% GCT), is the most stable phase during gait, and all these parameters represent the ability of the patient to transfer their body weight to the affected limb [62]. Our findings, similar to previous studies in RA patients, showed a significant increase in double support and a reduction in the swing phase [14]. This suggests that spending more time on both feet could be an adaptive approach to increase stability and reduce pain during gait.

### Gait variability

Analysis of gait variability is a clinically relevant parameter in the evaluation of gait and responses to interventions and is a viable option for the quantitative evaluation of gait stability [21]. To our knowledge, gait variability has never been investigated in people with PsA or other populations with foot involvement associated with arthritic joint disease. In our study, the mean stride time variability was higher in the PsA group (4.49 ± 3.56%) than in the control group (2.32 ± 0.72%) and above the normative values reported for stride time variability (1.1 to 2.6%) [49], indicating increased gait instability. This is consistent with novel findings from a recent study that reported an increased risk of falling in people with PsA [33]. Increased gait variability and instability could be ascribed to pain, muscle weakness, restricted range of motion, and a decrease in proprioception caused by inflammation in the foot joints and the surrounding structures [20]. However, there were no significant correlations between foot pain and stride time variability. Findings from a recent study reporting a significant alteration of static and dynamic balance in people with PsA also showed that there were no correlations between balance parameters, foot pain and foot function [32]. This suggests that pain may not be a determinant of gait variability and that this metric could be accepted as an independent gait parameter that should be assessed systematically in people with PsA. However, this needs to be confirmed in larger and longitudinal studies. Further studies are also needed to investigate the involvement of muscle weakness, reduced range of motion, and alterations of the proprioceptive system in gait variability in people with PsA.

This study showed that the disease duration and CRP levels were not correlated with self-reported foot pain and function, which is consistent with results from a previous study conducted in people with spondyloarthritis [63]. On the other hand, gait spatiotemporal parameters, especially gait speed, were strongly correlated with these clinical outcomes. It would be relevant to investigate these associations in larger and longitudinal studies and to link gait parameters to clinically relevant domains in PsA as determined by the GRAPPA [64].

Body-worn IMUs for gait analysis are more than ever used in clinical assessment and clinical studies in several neurological diseases, such as Parkinson’s disease, stroke, and multiple sclerosis [65–67]. These systems are easy to use, time- and cost-effective, do not require special equipment or expertise, and could be used in different settings. In addition, recent evidence suggests that they could accurately and reliably measure STPs in people with axial spondyloarthritis and PsA [23, 24]. This study suggests that body-worn IMUs could be useful to obtain an objective measure of functional mobility in people with PsA.

There are some limitations to this study. First, given the small sample size and the uneven distribution of males and females in our study sample, the findings cannot be generalized to the population. Second, the patients were included based on their subjective perception of foot involvement. Although from a clinical perspective, the patients’ perception of pain and disability is a vital criterion, adding ultrasonography/MRI data to confirm the presence of enthesopathy, tendinopathy, synovitis, and/or bone erosions would have given more insight into the severity of foot involvement. Third, CRP levels were documented from the participant’s clinical records, which led to missing data and a delay (up to 3 months in a few participants) between CRP level assessment and data collection. Moreover, important clinical domains, including disease activity, skin disease activity, and fatigue, were not assessed which could significantly limit the proper description of the study cohort. Additionally, it is important to mention that gait variability was assessed over a 10-m distance. Ideally, future studies should consider longer distances while assessing this metric. Finally, the presence or absence of foot deformity was recorded in a qualitative manner (presence/absence). Using standardized tools such as the foot posture index [68] could have been more relevant to ensure comparability between studies.

## Conclusion

Foot pain and disability have been reported to be important manifestations of PsA. This was confirmed in this study since severe levels of foot-related disability were reported despite the use of DMARD/biological therapy in more than 80 % of the patients. Disability was further demonstrated through the objective assessment of foot function. The findings showed that STPs obtained from IMUs during a standardized 10-m walk test were significantly altered and that there were strong correlations between pain, disability levels, and STPs. In addition, this study demonstrated for the first time increased gait variability in people with PsA which was not correlated with pain levels. This suggests that instability during gait in PsA could be independent of foot pain and that it should be further assessed in larger studies. The findings of this study add important information on gait in people with PsA, a population for which research on gait and posture is scarce.

## Data Availability

The dataset used and analyzed during the current study is available from walha.roua@usherbrooke.ca on reasonable request.

## Abbreviations

BMI: Body mass index
CRP: C reactive protein
CV: Coefficient of variation
DMARD: Disease-modifying anti-rheumatic drugs
FFI: Foot function index
GCT: Gait cycle time
MCID: Minimal clinically important difference
NRC: Numerical rating scale
PsA: Psoriatic arthritis
STPs: Spatiotemporal parameters
10MWT: 10-m walk test
